# Effect of Sn Doping on Pd Electro-Catalysts for Enhanced Electro-Catalytic Activity towards Methanol and Ethanol Electro-Oxidation in Direct Alcohol Fuel Cells

**DOI:** 10.3390/nano11102725

**Published:** 2021-10-15

**Authors:** Cyril Tlou Selepe, Sandile Surprise Gwebu, Thabo Matthews, Tebogo Abigail Mashola, Ludwe Luther Sikeyi, Memory Zikhali, Nobanathi Wendy Maxakato

**Affiliations:** 1 Department of Chemical Sciences, University of Johannesburg, Doornfontein, Johannesburg 2028, South Africa; cyrilkatlego15@gmail.com (C.T.S.); surprisesgwebu@gmail.com (S.S.G.); matthewsthabo@gmail.com (T.M.); tebogail@gmail.com (T.A.M.); zicaliememo@gmail.com (M.Z.); 2 Molecular Sciences Institute, School of Chemistry, University of the Witwatersrand, Braamfontein, Johannesburg 2050, South Africa; ludweluthersikeyi@gmail.com

**Keywords:** carbon nano-onions, electro-catalyst, fuel cells, palladium, tin, methanol, ethanol

## Abstract

Carbon nano-onions (CNOs) were successfully synthesized by employing the flame pyrolysis (FP) method, using flaxseed oil as a carbon source. The alcohol reduction method was used to prepare Pd/CNOs and Pd-Sn/CNOs electro-catalysts, with ethylene glycol as the solvent and reduction agent. The metal-nanoparticles were supported on the CNO surface without adjusting the pH of the solution. High-resolution transmission electron microscopy (HRTEM) images reveal CNOs with concentric graphite ring morphology, and also PdSn nanoparticles supported on the CNOs. X-ray diffractometry (XRD) patterns confirm that CNOs are amorphous and show the characteristic diffraction peaks of Pd. There is a shifting of Pd diffraction peaks to lower angles upon the addition of Sn compared to Pd/CNOs. X-ray photoelectron spectroscopy (XPS) results also confirm the doping of Pd with Sn to form a PdSn alloy. Fourier transform infrared spectroscopy (FTIR) displays oxygen, hydroxyl, carboxyl, and carbonyl, which facilitates the dispersion of Pd and Sn nanoparticles. Raman spectrum displays two prominent peaks of carbonaceous materials which correspond to the D and G bands. The Pd-Sn/CNOs electro-catalyst demonstrates improved electro-oxidation of methanol and ethanol performance compared to Pd/CNOs and commercial Pd/C electro-catalysts under alkaline conditions.

## 1. Introduction

With the growing energy crisis and environmental pollution over the last few decades, fuel cell technology has gained more attention [1]. A fuel cell is an electrochemical device which can transform chemical energy stored in a fuel into electrical energy. Fuel cells exhibit high efficiency of energy conversion and their electro-catalytic reactions produce minimal pollution relative to the polluting nature of combustion engines. Mostly the by-products are waste heat and water, which are environmentally acceptable [2]. Fuel cells are classed based on the type of electrolyte they utilize, which determines the operating temperature and catalyst that is appropriate for those conditions. Among various types of fuel cells, direct alcohol fuel cells (DAFCs) are the most attractive because they use liquid and renewable alcohol as fuel. They are recognized as green energy producers capable of transforming renewable sources into power by feeding liquid fuels straight to the anode [3].

Ethanol and methanol are the most utilized alcohols in DAFCs, due to low cost, easy handling and storage, and high energy density [4]. However, the methanol crossover effect—carbon monoxide (CO) poisoning of the catalyst and activation of the breaking of the C-C bond from ethanol at lower temperatures—is a major concern. The performance of a DAFC depends on catalyst activity and its lifetime. An electro-catalyst is a type of catalyst that speeds up the rate of an electrochemical reaction occurring on an electrode surface without being consumed during the process [5]. The Pt catalyst has excellent catalytic efficiency. However, it is costly, which hinders the commercialization of fuel cells [6]. There is a need to minimize the use of Pt and develop new catalysts to improve alcohol oxidation reactions. Over the years, palladium-based electro-catalysts have emerged as strong candidates to replace platinum catalysts. Pd-based catalysts demonstrate more activity in alkaline mediums and better resistance to CO than Pt-based catalysts [7,8]. Despite Pd possessing advantages over Pt-based catalysts for DAFCs, there are issues such as to enhance oxidation kinetics and improve antipoisoning capabilities which are associated with Pd-based catalysts. Some reports have shown that combining Pd with synergistic secondary metal oxides (e.g., CeO_2_) or palladium-based bimetallic or multimetallic alloy nanostructures (e.g., PdPt and PdPtNi) increases its electro-catalytic activity compared with its Pd counterpart [9,10]. For example, L. Nan et al. alloyed Pd with tin oxide (SnO_2_), demonstrating a reliable approach to improve the electro-catalytic activity of DAFCs [10]. SnO_2_ is a promising candidate because it supplies abundant OH species at the interface sites of the catalyst surface to remove CO intermediates [11]. Furthermore, being a strong metal-metal oxide interaction, it allows for uniform dispersion of metal nanoparticles as well as modification of a metal’s electronic state.

Exploring robust support materials is another feasible strategy to facilitate the alcohol oxidation reaction (AOR) performance of Pd-based catalysts; this strategy further improves the stability and utilization efficiency of metals [12]. Different types of carbon nanostructures have been utilized as catalyst supports which facilitate mass diffusion and prevent agglomeration and leaching of the metallic nanoparticles [13]. Pt- and Pd-based NPs supported on porous carbon nanomaterials are still commonly used as electro-catalysts. However, the corrosion of carbon supports in acid or alkaline conditions leads to the aggregation and leaching of Pd metal; hence, the activity of the catalyst decreases as a result of corrosion [14]. To avoid these fundamental issues, carbon nano-onions (CNOs)—zero-dimensional carbon with high porous structure and excellent electrical conductivity—have been used as support for electro-catalysts [15].

CNOs are a recent addition to the nanostructured carbon materials family; they are spherical particles with concentric shells of graphitic carbon that are typically 4–25 nm in diameter [16]. Due to their unique physical form of a shell-shaped structure, CNOs exhibit extreme surface areas and excellent tribological behavior, among other remarkable features. These characteristics have caused CNO particles to be widely researched for applications in various fields [17,18,19]. CNOs have a distinctive morphology, with concentric graphite rings and multiple edge planes that can be used as anchoring sites for metal nanoparticles. Dispersion of metal nanoparticles is aided by functionalizing the edge carbon atoms, which allows for increased particle loading [19]. CNOs have a large surface area, excellent Pd support, and high palladium dispersion, resulting in smaller particle sizes [20]. The production costs can be minimized by lowering the amount of palladium used. In this work, Pd and PdSn nanoparticles supported on carbon nano-onions were synthesized using the alcohol reduction method. Sn was introduced to supply abundant OH species at the interface sites to remove CO intermediates from the catalyst surface. This study aimed to develop catalyst support material for the enhancement of catalytic activity and stability of the electro-catalyst for alcohol oxidation reactions (AOR) in alkaline media.

## 2. Materials and Methods

### 2.1. Materials and Reagents

Methanol (CH_3_OH) 99.8%, Nafion 117 solution, palladium (II) chloride (PdCl_2_) 59–60%, tin(II) chloride (SnCl_2_) 98%, and potassium bromide (KBr) ˃99.9% were purchased from Sigma Aldrich (St. Louis, MO, USA). Ethanol (C_2_H_5_OH) ˃99.9%, sulphuric acid (H_2_SO_4_) 95.0–97.0%, hydrochloric acid (HCl) 24–26%, and sodium hydroxide pellets (NaOH) 98% were supplied by Honeywell (Midrand, South Africa) and SRL Chemicals (Maharashtra, India). Potassium hydroxide pellets (KOH) 85% were obtained from Merck Chemicals (Germiston, South Africa). Flaxseed oil and wick were purchased from Checkers and Pick n Pay, respectively (Johannesburg, South Africa). All reagents were of analytical grade and used as received without further purification. De-ionized water was used throughout to prepare solutions.

### 2.2. Synthesis of Carbon Nano-Onions (CNOs)

In this method, 50 mL of flaxseed oil was transferred into a 250 mL beaker. The wick was immersed into the beaker containing flaxseed oil. The oil-filled beaker was mounted underneath each brass collecting plate at an optimal distance of 40 mm between the nozzle and each plate. The experiment was conducted with a 30 mm distance between the top of each collecting plate. Under ambient air conditions, a wick soaked in oil was ignited to create a flame. Finally, the prepared carbon nano-onions were collected on a brass collecting plate (BCP) for 6 h [21].

### 2.3. Functionalization of CNOs

A 1.00 g amount of CNOs was dispersed in a 160 mL mixture of 2 M HNO_3_ and 2 M H_2_SO_4_ (1:3) in a 250 mL round-bottom flask. The reaction mixture was refluxed for 4 h to introduce carboxylic acid functional groups. The functionalized carbon nanomaterial was filtered and subsequently placed in a vacuum oven at 60 °C overnight to dry [22].

### 2.4. Synthesis of Pd/CNO and Pd-Sn/CNO Electro-Catalysts

A 1.00 g amount of CNOs, 83 mg PdCl_2_, and 42 mg SnCl_2_ were dispersed in 75 mL of ethylene glycol solution and ultrasonicated for 30 min. The mixture was refluxed for 4 h under magnetic stirring to allow for the reduction of the metal precursors. The resulting black suspension was filtered, washed with deionized water, and dried overnight at 60 °C to obtain Pd/CNO and Pd-Sn/CNO electro-catalysts.

### 2.5. Characterization of Nanomaterials

#### 2.5.1. Physicochemical Characterization

A Jeol JEM-2100F Electron Microscope instrument equipped with a LaB6 source was used to perform high-resolution transmission electron microscopic (HRTEM, JEOL, Tokyo, Japan) studies at an increasing speed voltage of 200 kV was conducted to study the internal morphology of synthesized nanomaterials. ZEISS-AURIGA field emissions scanning electron microscopy (FESEM, Zeiss Auriga, Carl Zeiss, Jena, Germany), 3 kV acceleration voltage was used to investigate the external morphology of the prepared nanomaterials. Fourier transform infrared spectroscopy (FTIR, PerkinElmer, Inc., Shelton, CT, USA) was performed using Model PerkinElmer Spectrum 100 to measure the infrared spectra of the carbon nano-onion supports and Pd/CNO and Pd-Sn/CNO electro-catalysts. In the frequency range of 500–4000 cm^−1^, the samples were scanned as solids. Raman spectra were recorded with a T64000 series II triple spectrometer system from HORIBA scientific, Jobin Yvon Technology, using Lab Spec 5 software and 514.3 nm laser line of a rational Innova^®^ 70C arrangement Ar + laser (spot estimate ~2 μm) with a determination of 2 cm^−1^ in the range 200–1800 cm^−1^. PANayltical X’Pert Pro powder diffractometer instrument was used to obtain the Powder X-ray diffraction (P-XRD, PANalytical BV, Almelo, The Netherlands) measurements of CNO, Pd/CNO, and Pd-Sn/CNO nanomaterials. Measurements were performed in the 2θ range from 5 to 90° with Cu Kα radiation (λ = 0.15405 nm) at 40 kV and 40 mA operational conditions. Brunauer–Emmett–Teller (BET, Micromeritics TriStar II Plus, Berlin, Germany) was used for surface area analysis. Inductively coupled plasma ICap 6000 series (Thermo Scientific, Johannesburg, South Africa) was used to evaluate the actual metal loading in electro-catalysts. X-ray photoelectron spectroscopy (model Kratos Axis Ultra DLD, Kratos Analytical, Inc., Manchester, UK) (XPS) was utilized to study the elemental composition and electronic state of palladium and tin within the carbon nano-onions using an Al (monochromatic) anode, furnished with a charge neutralizer.

#### 2.5.2. Electrochemical Characterization

##### Preparation of Catalyst Ink and Modified Electrode

A 10 mg amount of as-synthesized electro-catalysts was dispersed in 2 mL ethanol with 5 µL of Nafion solution to make the catalyst ink. The catalyst ink solution was sonicated for 15 min before 15 µL was drop-coated on a dried pre-treated glassy carbon electrode (GCE). The GCE was polished on Micro cloth TM (Buehler, Lake Bluff, IL, USA) with 1.0, 0.3, and 0.05 µm alumina slurries in decreasing order prior to drop coating. This was sonicated with double-distilled water to remove free particles from the micro cloth.

##### Electrochemical Measurements

Electrochemical investigations were conducted using Ivium Technologies Compactstat.h standard and a three-electrode system. The potentials of the working electrode were determined using the reference (Ag/AgCl) and counter (Pt wire) electrodes, which were both saturated with 3 M NaCl. During the electrochemical measurements, the working surface area of the modified GCE was 0.07 cm^−2^. The alkaline electrolyte solution was purged with argon 15 min before the electrochemical experiments. The chronoamperometry experiment was conducted while the solution was agitated under magnetic stirring at 50 rpm to keep the solution near the electrode homogeneous. All the results were compared to a commercial 10 wt% Pd/C electro-catalyst that was studied under the same conditions.

## 3. Results and Discussion

### 3.1. Physicochemical Characterization of Synthesized Materials

#### 3.1.1. Raman Spectroscopy

**Figure 1:**
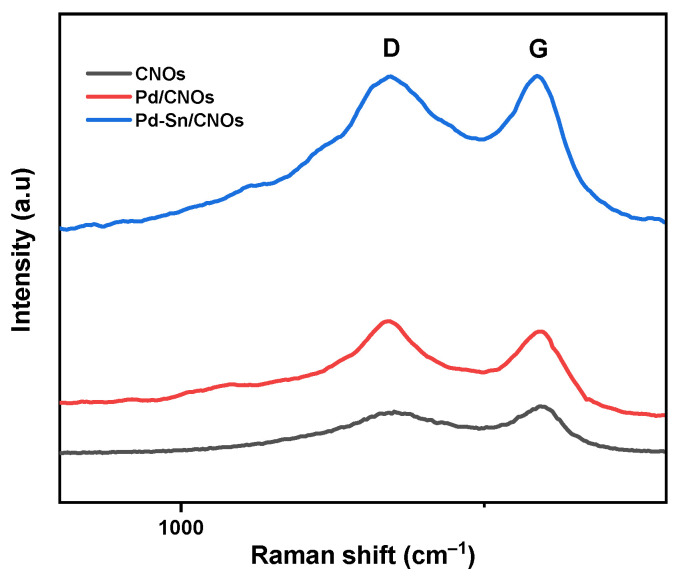
Raman spectra of CNO support material and Pd/CNO and Pd-Sn/CNO electro-catalysts.

The Raman spectra of carbon nano-onions (CNOs), Pd/CNOs, and Pd-Sn/CNOs are depicted in Figure 1. As expected for carbonaceous materials, all Raman spectra display two prominent peaks which correspond to D and G bands. The disordered carbon peak, caused by C–C vibrations and associated with the disruption of the sp^2^ hybridized carbon bonds, is responsible for the observed D-band. Dangling bonds, sp^3^ bonding, vacancies, and other carbon rings resulting from structural rearrangement all contribute to this [23,24]. The emergence of the shoulder around 1000 cm^−1^ is due to the existence of more sp^3^ hybridized carbons close to those found in the diamond-like amorphous material [24,25]. The G-band indicates a graphite peak. The in-plane vibration modes of the sp^2^ carbon bonds are attributed to the presence of this peak, which represents the order of graphitic carbons in the structural matrix of CNOs [26]. The ratio of the relative intensity of the D-band to that of the G-band (I_D_/I_G_) is used to quantify the degree of graphitization of CNOs produced. The degree of disorder in the form of sp^3^ hybridized carbons and the presence of shorter graphitic fragments are directly linked to the disorder density ratio. The I_D_/I_G_ value of CNOs is 0.85, denoting a high degree of graphitization within the CNO matrix. Meanwhile, the ratios of the Pd/CNO and Pd-Sn/CNO electro-catalysts were found to be 0.96 and 0.99, respectively. The higher I_D_/I_G_ values of the electro-catalysts compared to those of the CNO supports indicate that the incorporation of Pd and Sn into the CNOs caused defects in the CNO structure and lattice symmetry [27].

#### 3.1.2. High-Resolution Transmission Electron Microscopy (HR-TEM)

**Figure 2:**
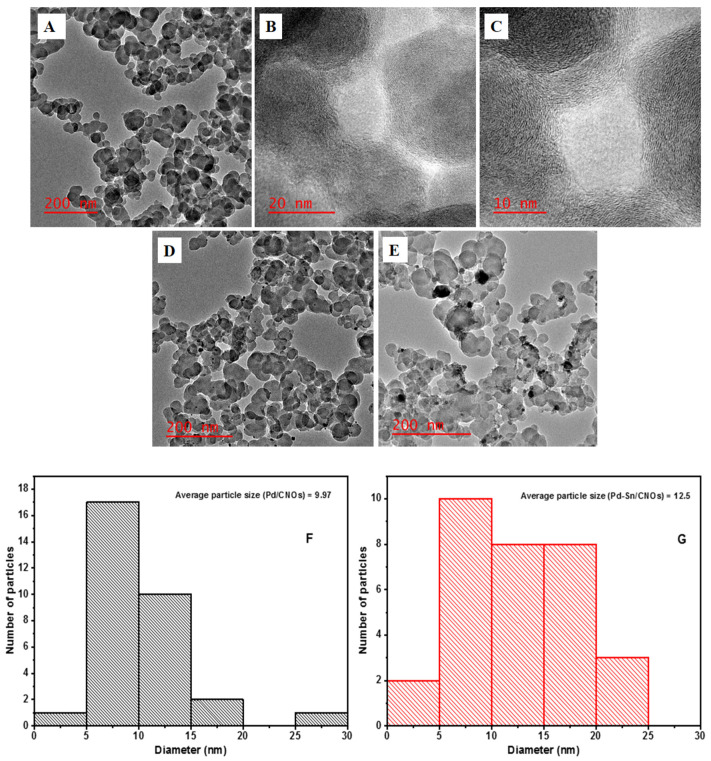
HR-TEM images of carbon nano-onion supports with different magnifications (**A**–**C**), Pd/CNO electro-catalysts, (**D**) and Pd-Sn/CNO electro-catalysts (**E**); average particle size of Pd NPs in Pd/CNOs (**F**) and Pd-Sn/CNOs (**G**).

The morphology of the as-prepared carbon nano-onions (CNOs) was investigated using HR-TEM; the micrographs are presented in Figure 2A–C. The HR-TEM micrographs reveal that the p-CNOs collected from brass collecting plates (BCP) are typical aggregated and agglomerated solid quasi-spherical-shaped CNOs. At the boundaries of the CNO particles, interpenetrating graphitic layers bind the quasi-spherical nanoparticles in an interconnected linkage [21]. With an interlayer spacing of 0.27 nm, concentric shells of graphitic layers can be seen, which are a distinctive feature of carbon nano-onions. The average size of the CNO nanoparticles is 42.30 nm. Short-range ordered concentric multi-layers were discovered in the p-CNOs (Figure 2B). The existence of disordered graphitic carbon is indicated by the lattice fringe spacing obtained [28]. The presence of nanoparticles on the CNO surface (Figure 2D) is an indication that palladium is successfully bonded on the carbon nanomaterial. The attachment of the metal is facilitated by the oxygen-containing groups found on the CNO surface. It can be observed that Pd nanoparticles (Pd NPs) are uniformly distributed on the surface of the CNOs, with an average particle size of 9.96 nm. There is no evidence of nanoparticle aggregation; this excellent dispersion may be associated with the high surface area and porosity of CNO support materials. Figure 2E displays HR-TEM images of Pd and Sn nanoparticles supported on the CNOs. It is worth noting from the figure that the metal nanoparticles are dispersed relatively well, with the average particle size of Pd being 12.50 nm. The image is consistent with the image shown in Figure 2D because Pd NPs can be observed. However, there is an appearance of new nanoparticles on the surface, which can be attributed to Sn NPs. The nanoparticles are grey, as opposed to the black Pd NPs, and are well-dispersed through the entire surface. This excellent dispersion is associated with various factors, such as the high surface area of CNOs, the functionalization of CNOs with a mixture of acids, and abundant OH species supplied by SnO_2_. The corresponding particle size histograms in Figure 2F,G show that the particle size increased by 2.54 nm upon the addition of tin. Figure S1 shows the selected area electron diffraction (SAED) pattern of the CNO supports (A) and Pd/CNOs (B) and Pd-Sn/CNO (C) electro-catalysts. These data provide further information about the crystallinity of the nanomaterials.

#### 3.1.3. Field Emission Scanning Electron Microscopy (FESEM)

**Figure 3:**
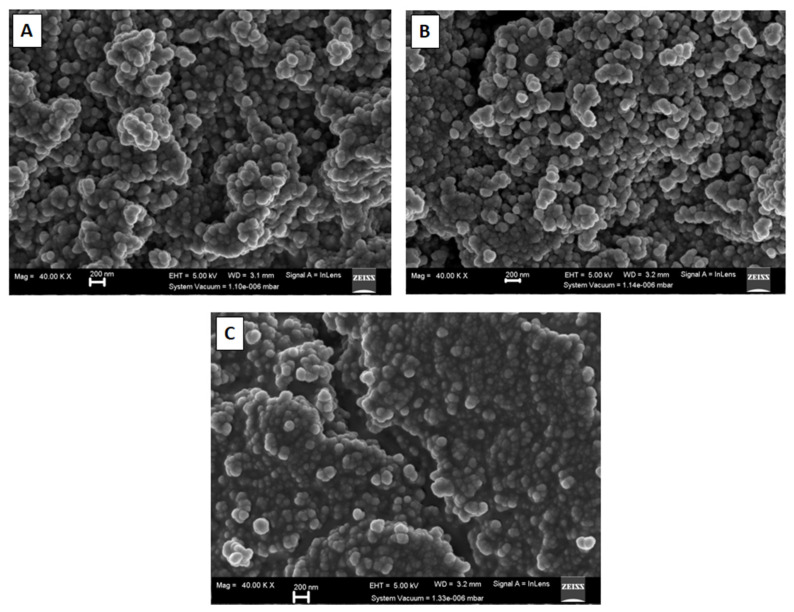
FESEM images of carbon nano-onion supports (**A**), Pd/CNO electro-catalysts (**B**), and Pd-Sn/CNOs electro-catalysts (**C**).

Field emission scanning electron microscopy (FESEM) images of carbon nano-onions supports and Pd/CNO and Pd-Sn/CNO electro-catalysts are depicted in Figure 3A–C. It is evidently clear that the images display the quasi-spherical morphology of CNOs, with aggregation of nanoparticles produced during the pyrolysis process. This morphology is similar to that previously described in the literature [29]. Energy dispersive X-ray analysis (EDX) was used to investigate the elemental compositions present in the carbon supports and electro-catalysts. The EDX spectra are shown in Figure S2, revealing the elemental composition of various elements present within the nanomaterials. Figure S2A–C illustrates that they are mainly composed of C and O, with Pd and Sn loadings detected under 10 wt% along with tiny traces of Al, Si, and Cl. Elemental mapping was done to investigate the elements present in the carbon nano-onion supports and Pd/CNO and Pd-Sn/CNO electro-catalysts. There is a uniform distribution of Pd and Sn atoms on the surface of the CNO supports. The elemental maps shown in Figure S3A–C indicate the formation of a PdSn alloy. Furthermore, the maps also depict that the nanoparticles have uniform sizes and are evenly distributed onto the CNO material.

#### 3.1.4. Fourier Transform Infrared Spectroscopy (FTIR)

**Figure 4:**
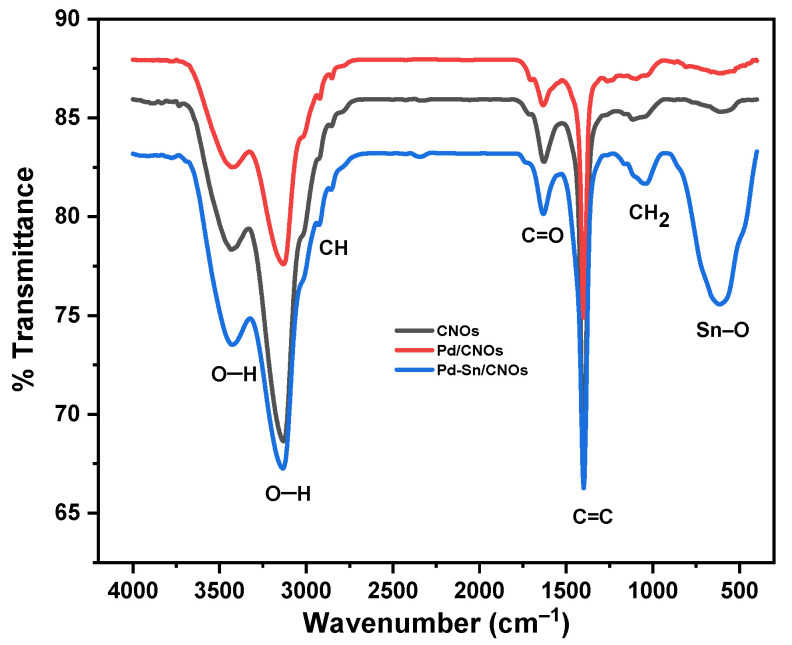
FTIR spectra of carbon nano-onion (CNO) supports and Pd/CNO and Pd-Sn/CNO electro-catalysts.

The existence of functional groups in the prepared CNOs, Pd/CNOs, and Pd-Sn/CNOs was evaluated by FTIR analysis (Figure 4). The CNOs exhibit vibrational bands at 3143 cm^−1^ and 3445 cm^−1^, ascribed to the OH vibrational stretch of the carboxylic group on the surface. Generally, the OH peak between 3000 and 3500 cm^−1^ is a broad peak. However, in this case, the peak is split into two; this is associated with the presence of alcohol O–H groups and water on the CNO surface [30]. Vibrations at 2849 cm^−1^ and 1085 cm^−1^ correspond to the aliphatic –CH and –CH_2_ groups, respectively. The vibration at 1636 cm^−1^ represents the C=O carbonyl group. The vibration at 1403 cm^−1^ corresponds to C=C stretching due to aromatic rings or an alkene functional group. The presence of carboxylic and hydroxyl groups on the CNO surface is confirmed by these peaks. These functional groups are responsible for the high hydrophilicity and dispersibility of the as-synthesized sample. The FTIR spectra of the Pd/CNO and Pd-Sn/CNO electro-catalysts are consistent with the results observed on the CNO spectrum, with the same functional groups observed. The attachment of the metal nanoparticles is promoted by the oxygen-containing species on the CNO surface [31,32].

#### 3.1.5. X-ray Diffractometry

**Figure 5:**
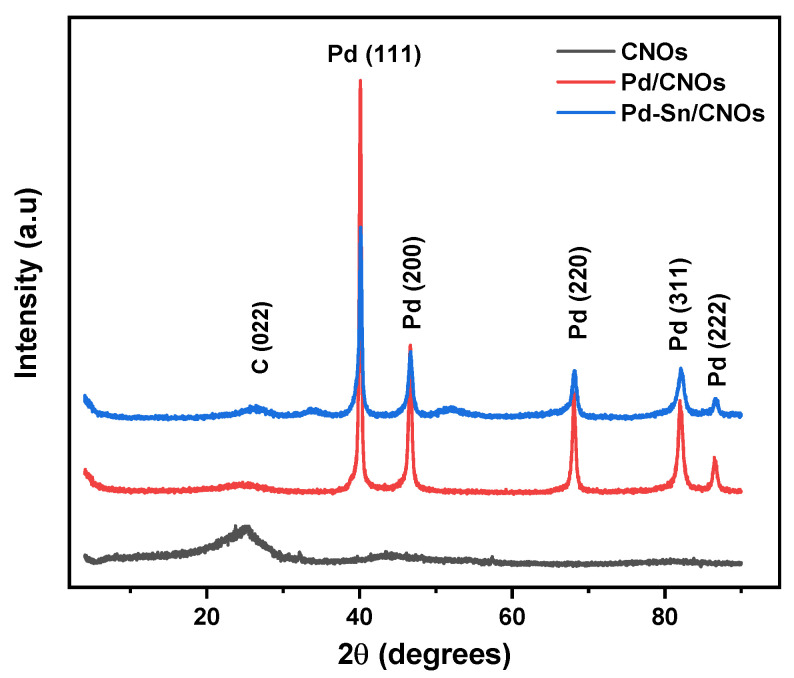
XRD patterns of carbon nano-onion (CNO) supports and Pd/CNO and Pd-Sn/CNO electro-catalysts.

XRD was performed to evaluate the crystal structure and phase purity of the CNOs, Pd/CNOs, and Pd-Sn/CNOs. Figure 5 shows the XRD pattern of the CNOs, which depicts a prominent graphitic peak at 2θ = 25.5°, corresponding to graphitic plane (002) reflection. The broadness of the (002) peak could be due to the CNOs’ small particle size and the stacked graphitic layers’ short domain order [33]. The broad peak between 2θ = 40° and 50° corresponds to the (100) and (101) reflections of the graphitic planes [34]. The XRD pattern of Pd-CNO nanocomposite clearly shows the characteristic diffraction peaks of Pd (111), (200), (220), (311), and (222) lattice planes at 2θ = 40°, 48°, 68°, 84°, and 87°, respectively. The existence of these new peaks in the Pd/CNO nanocomposite indicates a successful introduction of Pd nanoparticles. The intensity of the graphitic plane (002) of CNOs is too low due to the high peak intensity Pd peaks [35]. The Pd-Sn/CNO electro-catalyst exhibits broader diffraction peaks, which are the characteristic crystalline faces of Pd. The observed broad peaks indicate a decrease in particle size and an increase in the lattice parameter of palladium. The slight shift in the diffraction peaks indicates the formation of an alloy, specifically the formation of a solid palladium-tin alloy. The average particle size of Pd in the Pd-Sn-CNOs electro-catalyst was found to be 1.14 nm; this is almost half the particle size of Pd in the Pd/CNO electro-catalyst (2.32 nm). The lattice parameter of Pd in the Pd-Sn/CNOs was found to be 0.4876 nm; this is higher than that of Pd in the Pd/CNO electro-catalyst (0.3598 nm) due to the alloying of palladium with tin.

The average particle size of palladium in the electro-catalysts was calculated from the XRD peak of Pd (111) using the Debye-Scherer equation:

(1) $$D=\frac{0.9\lambda }{\beta \cos\theta }$$

where D is the particle size, 0.9 is a shape factor, θ is the angle of reflection, β is the peak at half maximum, and λ is the wavelength of the X-ray [36].

The lattice parameters of the electro-catalysts were calculated from XRD data using the equation below:

(2) $$\alpha =\frac{\left(2\sqrt{\lambda }\right)}{\sin\theta }$$

where λ the wavelength of the X-ray and θ is the angle of reflection [36].

#### 3.1.6. X-ray Photoelectron Spectroscopy (XPS)

**Figure 6:**
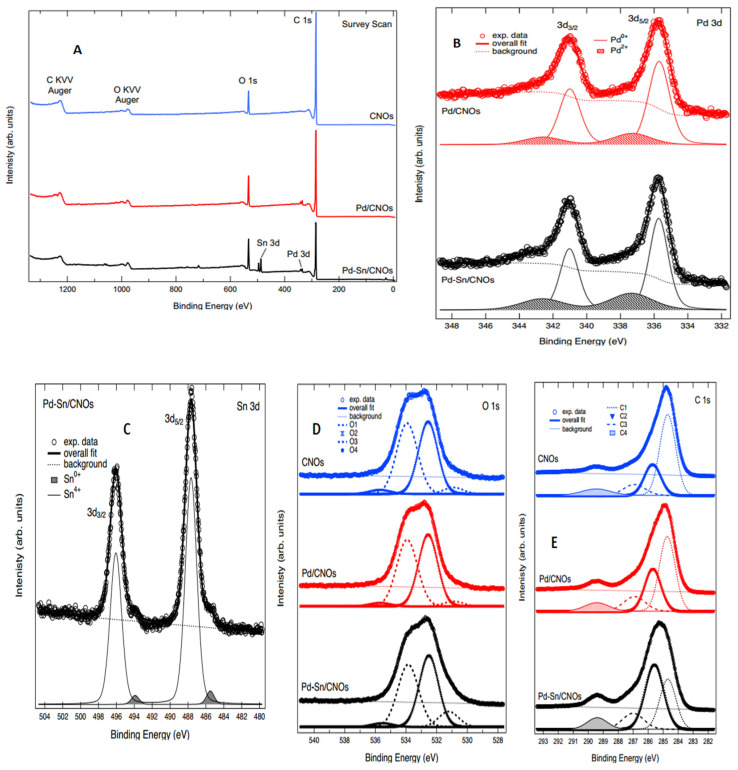
XPS spectra: (**A**) survey spectra for (**B**) Pd 3d for Pd/CNO and Pd-Sn/CNO electro-catalysts. (**C**) Sn 3d for Pd-Sn/CNO electro-catalyst; (**D**) O 1s for CNOs and Pd/CNO and Pd-Sn/CNO electro-catalysts; (**E**) C 1s for CNOs and Pd/CNO and Pd-Sn/CNO electro-catalysts.

XPS was used to evaluate the surface chemistry and electronic structures of the CNO series reported in Figure 6A. Only the major core levels and O KVV Auger lines have been labelled.

Figure 6B reports the Pd 3d XPS spectra for the Pd/CNOs and Pd-Sn/CNOs. Both spectra are composed of two main peaks ascribed to Pd 3d_5/2_ and 3d_3/2_ spin-orbit components, as labelled in the figure. The best fit to the experimental data was obtained by adding two spin-orbit doublets (both separated by a spin-orbit splitting of 5.3 eV, as per the relevant literature) and a Shirley-type background. The binding energy of these two components makes it possible to ascribe them to Pd^0+^ and Pd^2+^ oxidation states, which are appended to the figure [37]. The fit results with regards to binding energy (BE) and spectral weight of the components are reported in Table 1.

XPS was also used to study the oxidation states of Sn and Pd; Figure 6C shows the Sn 3d core level spectrum for the Pd-Sn/CNOs. The core level is composed of two main peaks corresponding to Sn 3d_5/2_ and 3d_3/2_ spin-orbit components, as labelled in the figure. Each of these two has a low-intensity shoulder on the low binding energy side of the main peak. The core level was therefore fitted with two spin-orbit components (separated by a spin-orbit splitting of 8.4 eV, as consistent with the relevant literature) and a Shirley background [38]. The binding energy of these components is consistent with ascribing them to 0^+^ and 4^+^ oxidation states for Sn ions in this sample, located at 485.51 eV and 487.66 eV, respectively, for 3d_5/2_. The comparison of the intensities shows that ~95% of the spectral intensity in this core level derives from the 4^+^ oxidation state [39].

The O 1s core level for the CNO samples is reported in Figure 6D. The best fit to the experimental data was obtained by adding four Voight line shape singlets (labelled O1, O2, O3, and O4 in the figure) to a Shirley background. The fitted components and the background are appended to the spectra, and the overall fit shows very good agreement with the experimental data. Based on binding energy and the relevant literature for similar compounds, we can ascribe the fitted components as follows: O1 to C–O bonds, O2 to C=O, O3 to C–O–H or C–O–C bonds, and O4 to O–C=O bonds [40]. The functional groups obtained from XPS are complemented by FTIR, indicating that the two techniques are in agreement. The fit results are reported in Table 2; they show very good agreement amongst the spectra.

Finally, Figure 6E displays the C 1s core level of the CNO samples. The line shape of the core level is structured, revealing the presence of several components contributing to it. The best fit to the experimental data was obtained using four Voigt line shape singlets and a Shirley background. The fitted components, labelled C1–C4, are appended to the figure together with the background. Based on the comparison of this line shape to the relevant literature, we can attribute the fitted components as follows: C1 to C=C bonds, C2 to C–C bonds, C3 to C–O bonds, and C4 to C=O bonds (which, given the broad line shape, could also incorporate the COOH contribution). Table 3 show the binding energies of the four components of C for CNO samples. The fit results with regards to binding energy (BE) are reported in Table 4; they show very good agreement amongst the spectra [41,42,43].

#### 3.1.7. Inductively Coupled Plasma Optical Emission Spectrometry (ICP-OES)

ICP-OES analysis was performed to determine the actual metal loading of the electro-catalysts. Table 5 shows the corresponding elemental composition. It can be seen that the measured total metal loads are lower than the theoretical total metal loads. The metal precursors (PdCl_2_ and SnCl_2_) were reduced to their respective metallic states using ethylene glycol. It is worth mentioning that, logically, the loading of Pd/CNOs should be higher than Pd-Sn/CNOs. However, this is not so because in Pd-Sn/CNOs, Pd was anchored on CNO supports and also in the Pd-Sn alloy, compared to Pd/CNOs where the anchoring site was only from CNOs.

#### 3.1.8. Brunauer-Emmett-Teller (BET) Surface Area Analysis

BET was performed to evaluate the surface area of the carbon nano-onion (CNO) supports and Pd/CNO and Pd-Sn/CNO electro-catalysts. The results, tabulated in a table below, indicate that the surface area decreased as the surface of the CNOs was modified with palladium, and further decreased after the addition of tin nanoparticles to the Pd/CNO electro-catalyst. Notably, the palladium and tin electro-catalysts reveal lower surface areas compared to the CNO supports, indicating coverage of pores by the Pd and Sn nanoparticles. The pore-size distribution (PSD) was investigated using the Barrett, Joyner, and Halenda (BJH) technique applied to the desorption branch [44]. The PSD revealed that the CNO supports have abundant mesopores, with pore diameter (39.5 nm). This mesoporisity of as-synthesized CNOs assisted in achieving a high metal nanoparticle dispersion. The existence of these mesopores for carbon nano-onion material is beneficial for improving rate kinetics and the quick dissemination of electrolyte ions [45]. Table 6 displays the summary of BET data for CNOs, Pd/CNOs and Pd-Sn/CNOs. In addition, N_2_ adsorption–desorption isotherms and BJH desorption pore-size distribution are presented in Figure S4A,B. These two plots support the information provided about the surface area analysis of the nanomaterials.

### 3.2. Application of Electro-Catalysts in Alcohol Fuel Oxidation Reactions

#### 3.2.1. Methanol Electro-Oxidation

**Figure 7:**
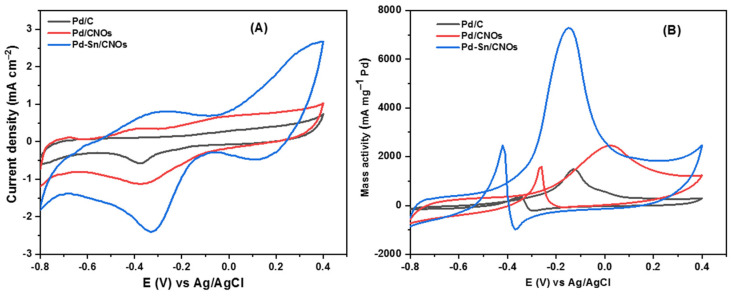

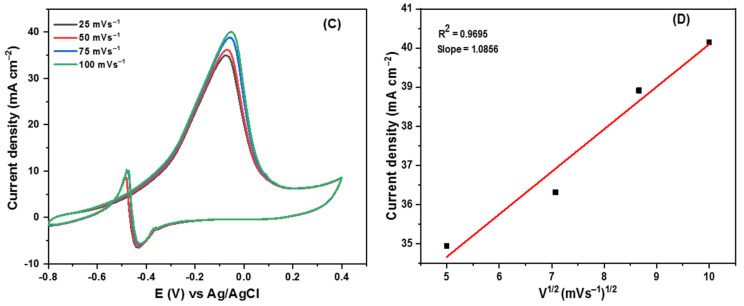
(**A**–**D**) Cyclic voltammogram of Pd/C, Pd/CNOs, and Pd-Sn/CNOs in 1 M KOH (**A**) and in 1 M KOH + 1 M CH_3_OH (**B**) solution at a scan rate of 50 mVs^−1^ at room temperature. Cyclic voltammogram of Pd-Sn/CNO electro-catalyst in 1 M KOH + 1 M CH_3_OH at different scan rates (**C**) and the plot of peak current density vs. square root of scan rates (**D**).

Figure 7A presents the CV curves of Pd/C, Pd/CNO, and Pd-Sn/CNO electro-catalysts on the GC electrode in 1 M KOH for the determination of electrochemical active surface area (EASA). In all CV curves, an OH^-^ adsorption peak was observed from −0.8 V to 0.4 V and a PdO reduction peak was observed from 0.4 V to −0.8 V. At lower potentials, the Pd-Sn/CNO electro-catalysts possess the highest oxygenated species reduction, facilitating alcohol oxidation [46]. The electrochemically active surface areas (EASA) of the electro-catalysts were calculated using these CV curves (Pd/C, Pd/CNOs, and Pd-Sn/CNOs) and the electro-catalysts’ EASA was determined using the following formula [47]:

(3) EASA=Qsl

where Q is coulombic charge obtained by integrating the area under the PdO reduction peak, s is the proportionality constant (405 µC cm^−2^), and l is the palladium loading (g cm^−2^).

The EASA values for the Pd/C, Pd/CNOs, and Pd-Sn/CNOs were found to be 142.94 cm^2^ mg^−1^, 168.32 cm^2^ mg^−1^, and 276.12 cm^2^ mg^−1^, respectively. The CV currents were normalized to the electrode surface area using these values. An enlarged EASA signifies the presence of more electrochemically active sites. The Pd-Sn/CNO electro-catalyst shows the highest EASA value, compared to the Pd/CNO and standard Pd/C electro-catalysts. This is due to the homogenous dispersion of PdSn metallic particles over the carbon nano-onion supports, indicating a strong metal–support interaction.

Two distinct forward and backward current peaks characterize alcohol oxidation reactions. The oxidation reaction is represented by the forward peak due to electro-oxidation of newly chemisorbed species resulting from adsorbed alcohol molecules. The elimination of intermediates that were only partially oxidized in the forward scan is represented by the backward peak. The catalytic activity for the electro-oxidation reactions of alcohol fuels is measured by the size of the forward peak [48,49,50]. As shown in Figure 7B, it is evident that electro-catalytic activity for methanol oxidation on the Pd-Sn/CNO electro-catalyst is greater than that of the Pd/CNO and Pd/C electro-catalysts. This may be due to the high surface area and higher palladium utilization provided by the support material, faster methanol adsorption, and availability of oxygen-containing species (OH^−^) in alkaline medium and provided by PdSn alloy. In an alkaline medium, the backward peak is smaller than the forward peak. This is because, in an alkaline medium, the removal of adsorbed carbonaceous species is easier due to the availability of oxygen-containing species (hydroxyl and carbonyl carbon), which allow faster methanol oxidation [51].

The Pd-Sn/CNO electro-catalyst outperformed the other two electro-catalysts as indicated by high current density, low potential, and stability. Figure 7C shows the cyclic voltammogram of the Pd-Sn/CNO electro-catalyst in 1 M KOH + 1 M CH_3_OH solution at different scan rates. As depicted in Figure 7C, the anodic peak current increases with increasing scan rates. However, it can be observed that with increasing scan rate, the anodic peak potentials slightly differ. A reversible relationship is indicated by the oxidation and reduction peaks for methanol [52]. This reversibility may be due to a sufficient charge transfer rate to keep the surface in equilibrium. A plot of peak current density and the square root of the scan rate is displayed in Figure 7D. The current density appears to be proportional to the square root of the scan rate. This means that the methanol oxidation on the Pd-Sn/CNO electro-catalyst on the electrode is regulated by a diffusion process to some extent. It has a slope of 1.0856, which is greater than the theoretical value of 0.5 for a diffusion-controlled reaction at the electrode surface [53].

**Figure 8:**
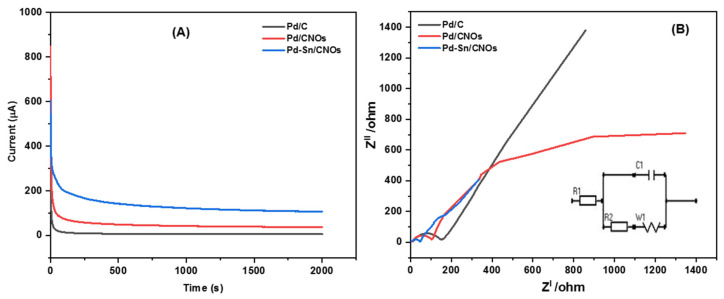
Chronoamperograms (**A**) and Nyquist plots with corresponding equivalent circuit (**B**) of Pd/C, Pd/CNO, and Pd-Sn/CNO electro-catalysts in 1 M KOH + 1 M CH_3_OH solution.

Figure 8A presents chronoamperometric (CA) curves of the Pd/C, Pd/CNO, and Pd-Sn/CNO electro-catalysts at a constant potential of 0.5 V for 2000 s. It can be observed from the CA curves that the initial current decay is very fast for all the electro-catalysts. This can be attributed to the adsorption of carbonaceous intermediates on the electro-catalyst surface during the entire reaction. However, the rate of current degradation for the Pd-Sn/CNO electro-catalyst is slower compared to the Pd/C and Pd/CNOs electro-catalysts. In the electro-oxidation of methanol, the Pd-Sn/CNO electro-catalyst displays higher stability and durability, as well as a lower poisoning rate, when compared to the other electro-catalysts. This indicates that the PdSn alloy provides oxygen-containing species on the surface that react with the poisoning intermediates, as the prepared Pd-Sn/CNO electro-catalyst exhibits a better current decrease than the others. After 500 s, the reaction reaches a stable current state. The chronoamperometry results are in line with the results obtained from cyclic voltammetry [54]. The plot of relative current versus time in Figure S5A helped to deduce the electro-catalytic activity retentions of the electro-catalysts. After 2000 s, the current lost for the Pd/C, Pd/CNO, and Pd-Sn/CNO electro-catalysts is 98.62%, 95.64%, and 82.2%, and the current retained is 1.38%, 4.36%, and 17.8%, respectively.

EIS was used to assess the catalytic efficiency of a Pd-Sn/CNO electro-catalyst for electro-oxidation of methanol in an alkaline electrolyte. The Nyquist impedance spectra of each anode electro-catalyst was recorded in the frequency range from 0.005 Hz to 1000 kHz (Figure 8B). The electrical equivalent circuit consists of solution resistance (R1), charge transfer resistance (R2), Warburg (W1), and circuit cell (C1). The radius of the impedance arc was used to study the charge transfer resistance for each electro-catalyst [55]. It is evidently clear that the semi-circle diameter of the Pd-Sn/CNO electro-catalyst is small in comparison with the other electro-catalysts. As a result, Pd-Sn/CNO electro-catalysts possess a decrease in charge transfer resistance, which results in a fast charge transfer rate. The order of the semi-circle diameters is Pd-Sn/CNOs ˂ Pd/CNOs ˂ Pd/C, indicating that the rate at which methanol was oxidized on the electrode surface was faster in the Pd-Sn/CNO modified electrode [56].

#### 3.2.2. Ethanol Electro-Oxidation

**Figure 9:**
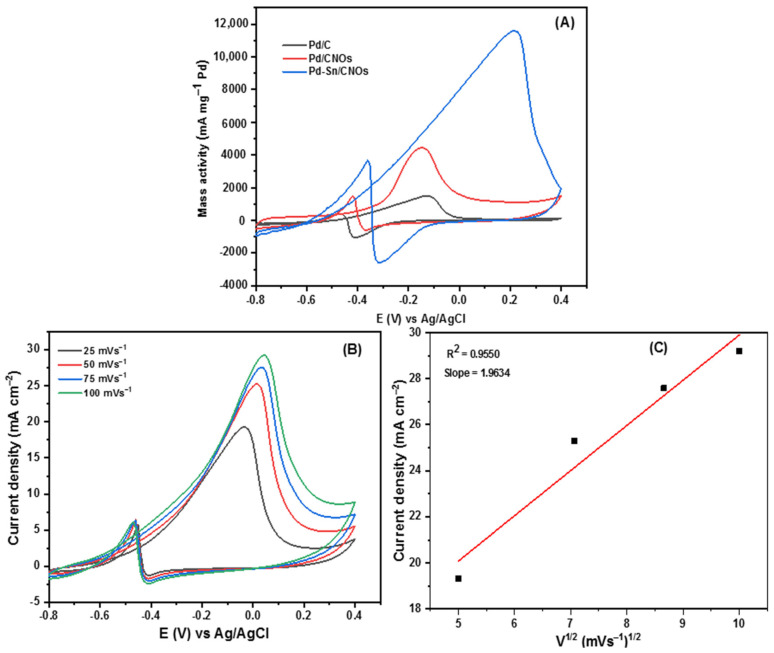
Cyclic voltammogram of Pd/C, Pd/CNOs, and Pd-Sn/CNOs in 1 M KOH + 1 M CH_3_CH_2_OH solution at a scan rate of 50 mVs^−1^ at room temperature (**A**). Cyclic voltammogram of Pd-Sn/CNOs electro-catalyst in 1 M KOH + 1 M CH_3_CH_2_OH at different scan rates (**B**) and the plot of peak current density vs. square root of scan rates (**C**).

Figure 9A shows the cyclic voltammograms of the Pd/C, Pd/CNO, and Pd-Sn/CNO electro-catalysts for ethanol electro-oxidation. The observed cyclic voltammograms display two distinct forward and backward current peaks, which are characteristic of anodic ethanol oxidation reactions. The electro-catalytic activity of the Pd-Sn/CNO electro-catalyst for ethanol oxidation is significantly higher than that of the commercial Pd/C and Pd/CNO electro-catalysts. This can be attributed to the high surface area and higher palladium utilization provided by the support material, faster ethanol adsorption, and availability of oxygen-containing species (OH) in an alkaline medium [57]. The magnitude of the backward peak is less than that of the forward peak in an alkaline medium. This is because the removal of adsorbed carbonaceous species is easier due to the availability of oxygen-containing species that facilitate quick ethanol oxidation [58,59,60].

Figure 9B presents the cyclic voltammogram of the Pd-Sn/CNO electro-catalyst at various scan rates from 25 to 100 mVs^−1^. To evaluate the electrochemical processes occurring at the modified glassy carbon electrode, the effect of scan rate on current density was investigated (adsorption or diffusion). As expected, the anodic peak current density is directly proportional to the square root of the scan rate. As the peak current density increases with the scan rate, the peak potential is drifting to more extreme potentials. The development of poisonous intermediates during the oxidation of ethanol may be linked to this change in potentials [61]. Despite the fact that poisonous species slow down the catalytic activity of the electro-catalyst, the Pd-Sn/CNO electro-catalyst exhibited a higher current density and stability in contrast to the other electro-catalysts. To better understand the processes occurring at the electrode surface, the peak current of each scan rate was plotted against the square root of the scan rate. The plot of the Pd-Sn/CNO electro-catalyst in Figure 9C does not have a perfect linear fit graph. This is mainly caused by electron transfer reactions occurring between the electrode and adsorbed molecules on the glassy carbon electrode during ethanol oxidation [62].

**Figure 10:**
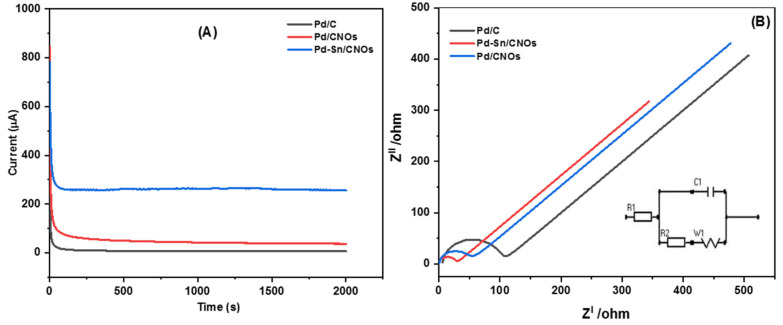
Chronoamperograms (**A**) and Nyquist plots with corresponding equivalent circuit (**B**) of Pd/C, Pd/CNO, and Pd-Sn/CNO electro-catalysts in 1 M KOH + 1 M CH_3_CH_2_OH solution.

In order to evaluate the stability of the ethanol oxidation reaction on all the catalysts, chronoamperometric (CA) measurements were taken for 2000 s at 0.5 V in a solution of 1 M KOH + 1 M CH_3_CH_2_OH shown in Figure 10A. The poisoning of the electro-catalysts causes a steady decrease in current density in all CA curves. During the electro-oxidation of ethanol, the formation of Pd oxides or hydroxides, as well as other intermediates adsorbed on the electro-catalysts, causes the decline [63,64]. The rate of current degradation for the Pd-Sn/CNO electro-catalyst is much slower than the Pd/CNO and Pd/C electro-catalysts. The residual current densities after 2000 s for the Pd/CNO and Pd-Sn/CNO electro-catalysts are higher than that for the commercial Pd/C electro-catalyst. This indicates high stability and durability for as-synthesized electro-catalysts compared to standard Pd-C. However, the Pd-Sn/CNO electro-catalyst is the most stable and durable electro-catalyst, because the Sn in the PdSn alloy provides oxygen-containing species that react with the poisoning intermediates, as seen by the slower current decay of the Pd-Sn/CNO electro-catalyst. The *y*-axis of the CA curves was normalized to convert it into a relative current (%) (Figure S5B) in order to further understand the stability and durability of the electro-catalysts. The current lost was calculated as 98.6%, 95.6%, and 72% for the Pd/C, Pd/CNO, and Pd-Sn/CNO electro-catalysts, respectively. The Pd-Sn/CNO electro-catalyst exhibited a high current retention (28%) compared to the other electro-catalysts.

Figure 10B illustrates the impedance spectra of the Pd/C, Pd/CNO, and Pd-Sn/CNO electro-catalysts and equivalent circuits. The latter consists of the solution resistance (R1), charge transfer resistance (R2), Warburg (W1), and cell (C1). For these electro-catalysts, semi-circles at different frequency regions can be observed. The Pd-Sn/CNO electro-catalyst has the smallest semi-circle diameter arc, indicating a smaller charge transfer resistance and leading to a fast charge transfer rate [65]. Therefore, the Pd-Sn/CNO electro-catalyst exhibited the best catalytic activity, compared to the Pd/C and Pd/CNO electro-catalysts.

## 4. Conclusions

Highly stable and electro-active carbon nano-onions supporting a palladium and tin alloy (Pd-Sn/CNOs) were prepared successfully. Carbon nano-onions were prepared by the flame pyrolysis method, using flaxseed oil as a carbon source. Thus, the PdSn alloy could be utilized at a smaller rate to counter the metal cost. Carbon nano-onions reduced the utilization of Pd and Sn while enhancing the capability of the electro-catalyst. Pd-Sn/CNOs with a high amount of alloying between Pd and Sn NPs demonstrate improved electro-catalytic activity. Chronoamperometry results also showed that the Pd-Sn/CNO electro-catalyst exhibited improved electro-catalytic stability and lower poisoning rate towards methanol and ethanol oxidation compared to the other electro-catalysts. The results obtained from EIS are in good agreement with the CV and CA results. The findings revealed that when particle dispersion and particle size are adjusted on a CNO support, the metal loading effect dominates electrochemical activity. The source of the CNO and the method of metal loading play a role in achieving high mass loading with regulated particle size and high dispersion. It was further revealed that Pd-Sn/CNO nanocomposites exhibit better electro-catalytic activity towards methanol and ethanol oxidation than Pd/CNOs and Pd/C in alkaline conditions and have great potential for applications in other electro-catalytic reactions.

## Figures and Tables

**Table 1:** Average crystal sizes and lattice parameters of Pd in the synthesized nanomaterials.

Electro-Catalysts	Pd in Pd/CNOs	Pd in Pd-Sn/CNOs
Average crystal size	2.32 nm	1.14 nm
Lattice parameter	0.3598	0.4876

**Table 2:** BEs (in eV) and relative percentage areas (in %) of the two components of the Pd 3d core level for CNO samples.

Electro-Catalyst	Pd^2+^ (%)	Pd^0+^ (%)
Pd-CNOs	80.5	19.5
Pd-Sn-CNOs	71.0	29.0

**Table 3:** Binding energies (in eV) of the four components of the C 1s core level for CNO samples.

Sample	C1 (eV)	C2 (eV)	C3 (eV)	C4 (eV)
CNOs	284.7	285.71	286.92	289.46
Pd-CNOs	284.67	285.70	286.97	289.67
Pd-Sn-CNOs	284.72	285.66	286.9	289.60

**Table 4:** Binding energies (in eV) of the four components of the O 1s core level for CNO samples.

Sample	O1 (eV)	O2 (eV)	O3 (eV)	O4 (eV)
CNOs	530.9	532.56	533.93	535.7
Pd-CNOs	530.91	532.56	533.97	535.65
Pd-Sn-CNOs	530.84	532.55	533.89	535.73

**Table 5:** Metal element loading of the synthesized electro-catalysts.

Electro-Catalyst	Pd (wt.%)	Sn (wt.%)	Actual Total Mass Content (wt.%)	Theoretical Total Mass Content (wt.%)
Pd/CNOs	8.20	0.06 *	8.26	10
Pd-Sn/CNOs	9.15	4.40	13.55	Pd (10) + Sn (5) = 15

* The value of Sn is negligible because it’s from an assay of Pd precursor.

**Table 6:** Summary of BET data for CNOs, Pd/CNOs, and Pd-Sn/CNOs.

Nanomaterial	BET Surface Area (m^2^/g)	Pore Volume (cm^3^/g)	Pore Size (nm)
CNO support	71.7872	0.71	39.5
Pd/CNO electro-catalyst	64.9401	0.75	46.3
Pd-Sn/CNO electro-catalyst	48.0655	0.71	59.4

## Data Availability

Data are available on request; please contact Nobanathi W. Maxakato, +27-83-513-4598, nmaxakato@uj.ac.za.

## Supplementary Materials

The following are available online at https://www.mdpi.com/article/10.3390/nano11102725/s1, Figure S1: Selected area electron diffraction pattern of carbon nano-onions (A), Pd/C (B) and Pd-Sn/CNOs (C) electro-catalysts. Figure S2: EDX spectrum of carbon nano-onions support (A), Pd/CNOs (B) and Pd-Sn/CNOs (C) electro-catalysts. Figure S3: The elemental mapping of carbon nano-onions support (D), Pd/CNOs (E) and Pd-Sn/CNOs (F) electro-catalysts. Figure S4: The N_2_ adsorption-desorption isotherms (A) and BJH desorption pore-size distribution (B). Figure S5: (A): display the chronoamperometric curve of Pd/C, Pd/CNOs and Pd-Sn/CNOs electro-catalysts in 1 M KOH + 1 M CH_3_OH solution and (B) in 1 M KOH + 1 M CH_3_CH_2_OH solution.
