# Supplementary material for: Effect of Sn Doping on Pd Electro-Catalysts for Enhanced Electro-Catalytic Activity towards Methanol and Ethanol Electro-Oxidation in Direct Alcohol Fuel Cells
Source: Nanomaterials (Basel). 2021 Oct 15;11(10):2725. doi: 10.3390/nano11102725 (PMC8537662; doi:10.3390/nano11102725)
Supplement: Supplementary file 1 [file nanomaterials-11-02725-s001.zip › nanomaterials-1377530-supplementary.pdf]

Supplementary materials

Effect of Sn Doping on Pd Electro-catalysts for Enhanced Electro-catalytic Activity Towards Methanol and Ethanol Electro-oxidation in Direct Alcohol Fuel Cells

Cyril. T. Selepe[1], Sandile. S. Gwebu[1], Thabo. Matthews[1], Tebogo. A. Mashola[1], Ludwe. L. Sikeyi[2], Memory. Zikhali[1] and Nobanathi. W. Maxakato[1]

[1] Department of Chemical Sciences, University of Johannesburg, Doornfontein 2028, South Africa.
[2] Molecular Sciences Institute, School of Chemistry, University of the Witwatersrand, Braamfontein 2050, South Africa;
* Correspondence: nmaxakato@uj.ac.za; Tel.: +27 (0) 11 559 6151

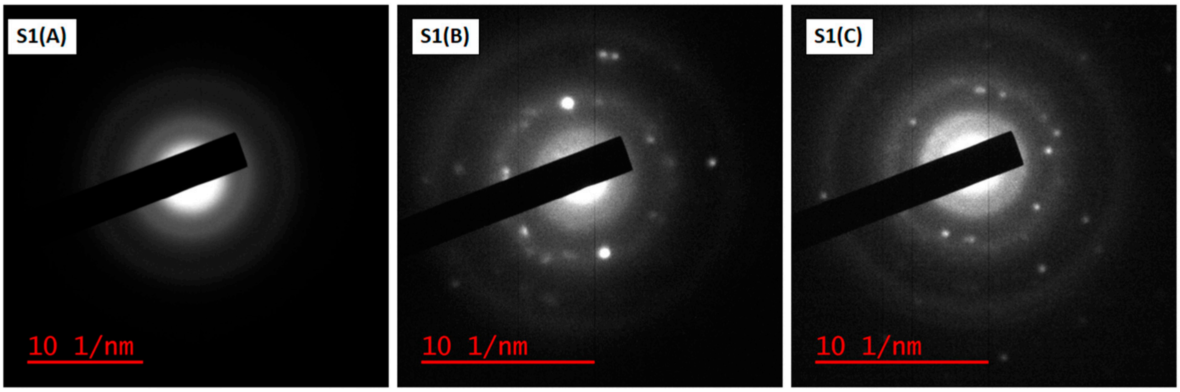

Figure S1 Shows selected area electron diffraction pattern of carbon nano-onions (A), Pd/C (B) and Pd-Sn/CNOs (C) electro-catalysts.

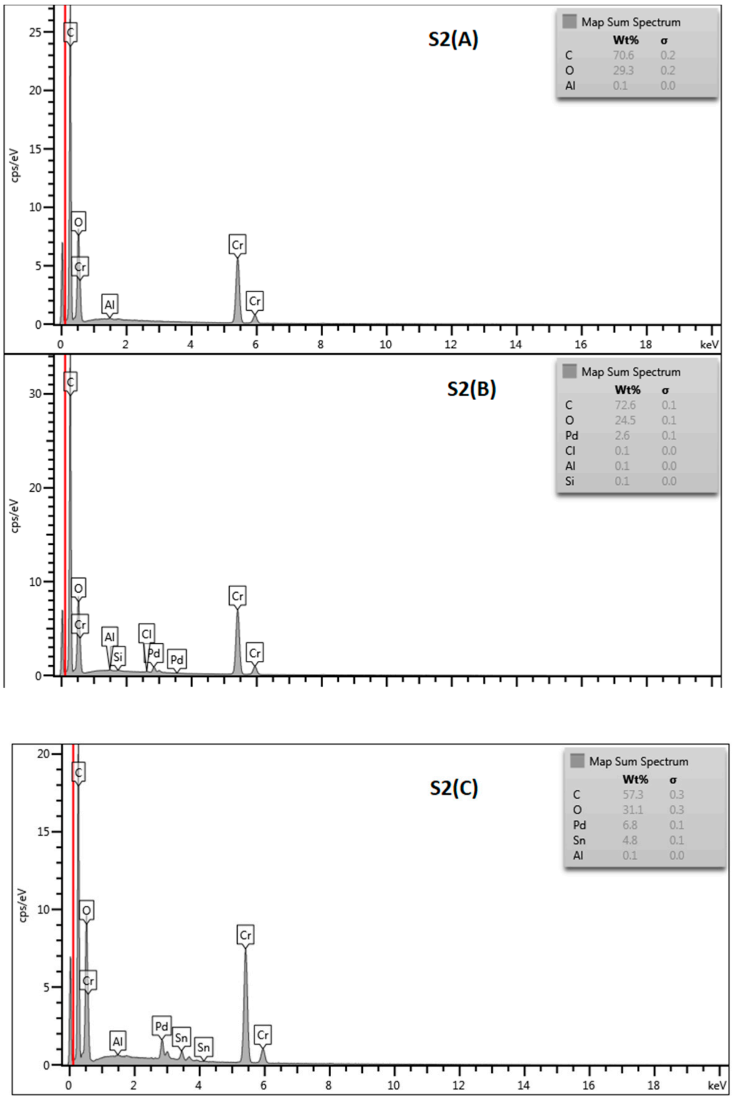

Figure S2: depicts the EDX spectrum of carbon nano-onions support (A), Pd/CNOs (B) and Pd-Sn/CNOs (C) electro-catalysts.

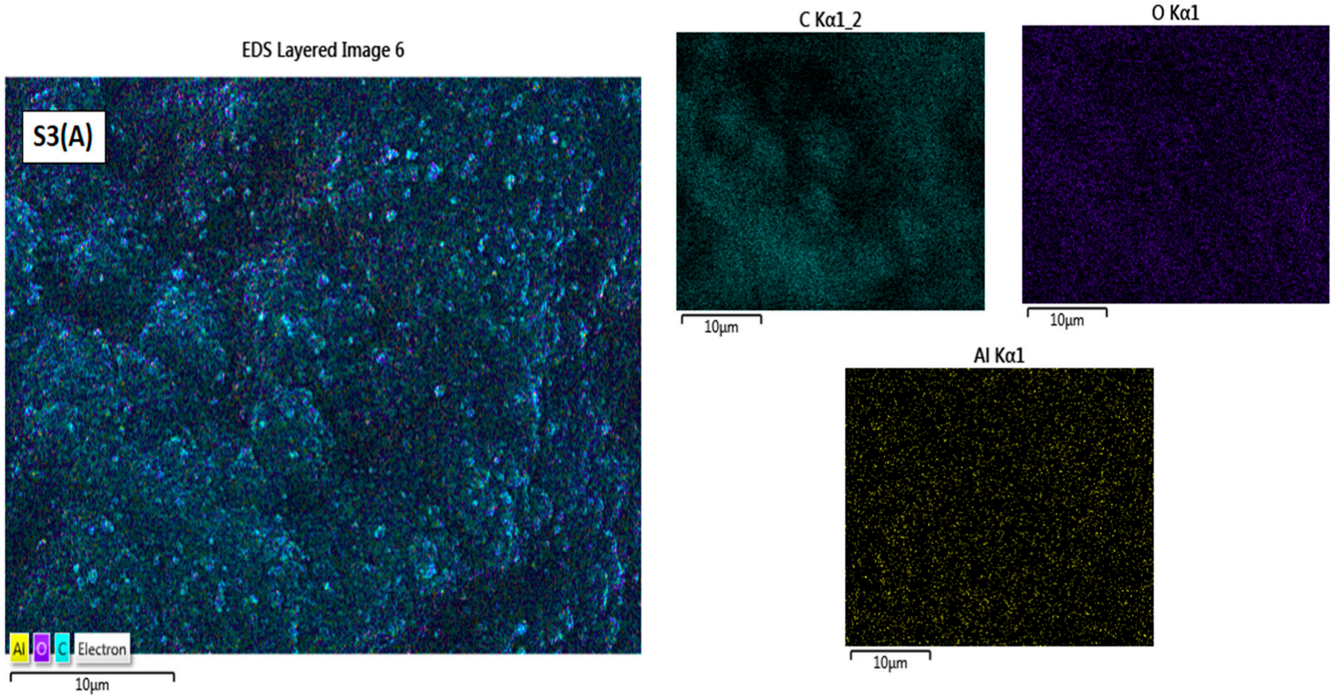
EDS Layered Image 6
S3(A)
Al
O
C
Electron
10µm
C Kα1_2
10µm
O Kα1
10µm
Al Kα1
10µm

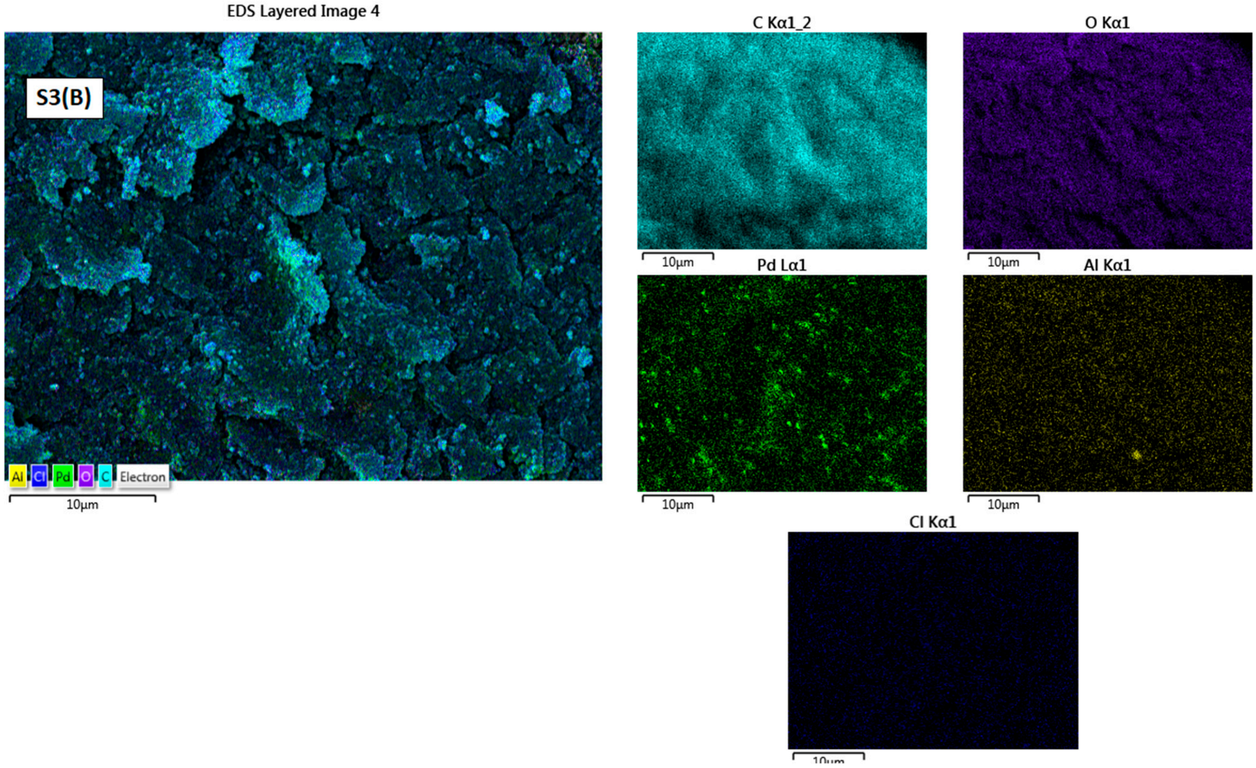
EDS Layered Image 4
S3(B)
Al
Cl
Pd
O
C
Electron
10µm
C Kα1_2
10µm
O Kα1
10µm
Pd Lα1
10µm
Al Kα1
10µm
Cl Kα1

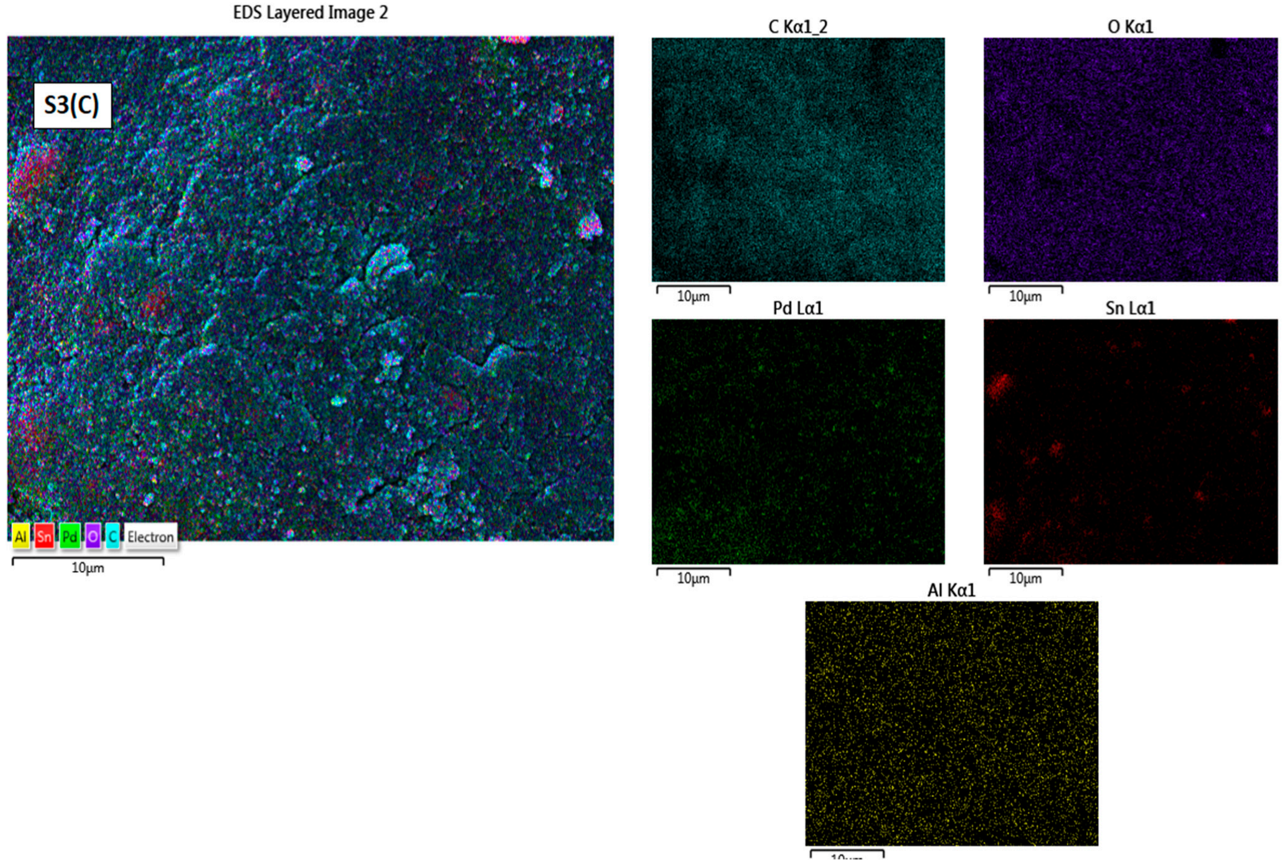

Figure S3: shows the elemental mapping of carbon nano-onions support (A), Pd/CNOs (B) and Pd-Sn/CNOs (C) electro-catalysts.

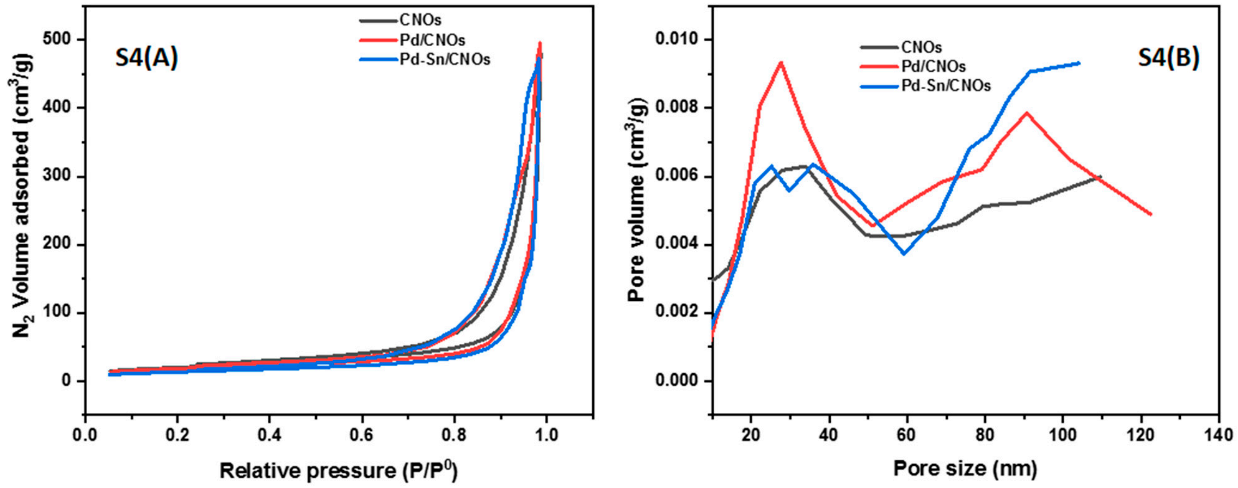

Figure S4: Shows the N_2 adsorption-desorption isotherms (A) and BJH desorption pore-size distribution (B).

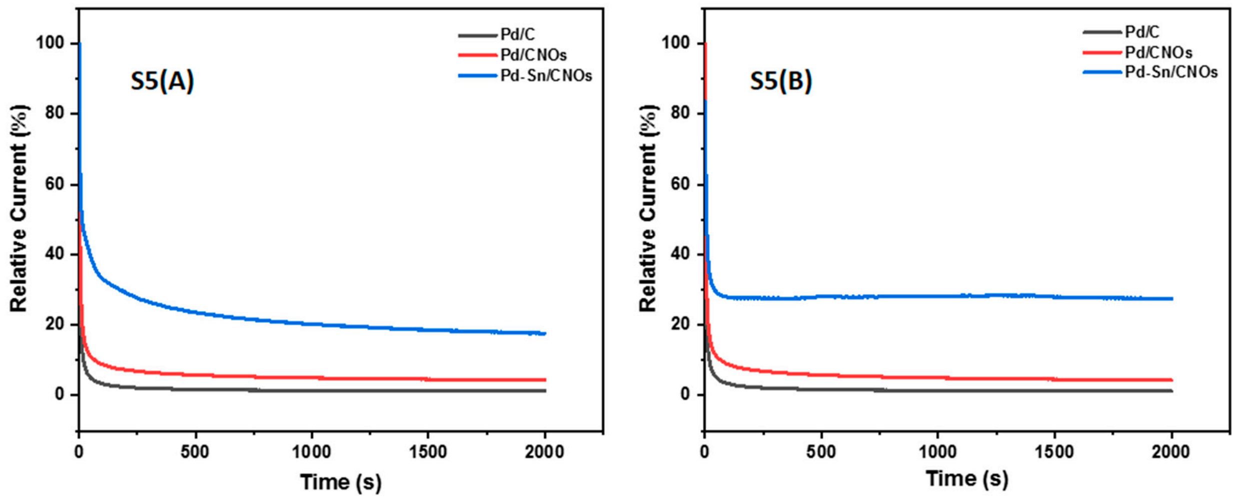

Figure S5 (A): display the chronoamperometric curve of Pd/C, Pd/CNOs and Pd-Sn/CNOs electro-catalysts in 1 M KOH + 1 M CH_3OH solution and (B) in 1 M KOH + 1 M CH_3CH_2OH solution.
